# Functional Outcomes after Selective Clamping in Robot-Assisted Partial Nephrectomy

**DOI:** 10.3390/jcm11195648

**Published:** 2022-09-25

**Authors:** Kiyoshi Takahara, Mamoru Kusaka, Takuhisa Nukaya, Masashi Takenaka, Kenji Zennami, Manabu Ichino, Hitomi Sasaki, Makoto Sumitomo, Ryoichi Shiroki

**Affiliations:** 1 Department of Urology, Fujita-Health University School of Medicine, Nagoya 470-1192, Japan; 2 Okazaki Medical Center, Department of Urology, Fujita Health University, Okazaki 444-0827, Japan

**Keywords:** robot-assisted partial nephrectomy, selective clamping, estimated glomerular filtration rate

## Abstract

This study aimed to assess the risks and benefits of selective clamping in robot-assisted partial nephrectomy (RAPN). We retrospectively analyzed 372 patients who had undergone RAPN at our hospital between July 2010 and March 2021. After propensity score matching between the full and selective clamping groups, perioperative outcomes and postoperative preservation ratio of the estimated glomerular filtration rate (eGFR) were compared at 6 and 12 months of follow-up. After propensity score matching, we evaluated 47 patients from each group. While no significant differences were observed in surgical time, warm ischemia time, or incidence rates of all grades of complications between the two cohorts, the estimated blood loss (EBL) was significantly lower in the full clamping group than in the selective clamping group (30 vs. 60, *p* = 0.046). However, no significant intergroup differences were observed in the postoperative preservation ratio of eGFR at 6 or 12 months of follow-up (full clamping 94.0% vs. selective clamping 92.7%, *p* = 0.509, and full clamping 92.0% vs. selective clamping 91.6%, *p* = 0.476, respectively). Selective clamping resulted in higher EBL rates than did full clamping in RAPN. However, selective clamping provided no renal functional advantage over full clamping in our propensity-score-matched cohort.

## 1. Introduction

Partial nephrectomy (PN) is the standard treatment for small renal tumors as the long-term oncological and functional outcomes are superior to those of radical nephrectomy [1,2]. According to a comprehensive systematic review and meta-analysis on PN, robot-assisted partial nephrectomy (RAPN) delivers mostly superior and at a minimum equivalent outcome compared to open and laparoscopic PN [3].

Multiple factors, including baseline function, amount of preserved parenchyma, and warm ischemia time (WIT), affect short- and long-term renal function after RAPN. Of these, WIT is considered a primary factor, and WIT > 25 min is particularly associated with a significant risk of new-onset stage IV chronic kidney disease [4].

Selective renal artery clamping is an alternative surgical technique to full clamping, that involves isolating and clamping only those renal arterial branches that directly supply the renal tumor. Selective clamping is performed over full clamping primarily due to better renal functional outcomes. Recently, several studies have evaluated the effectiveness and ability of performing selective clamping in RAPN [5,6,7,8,9,10,11,12,13,14,15,16,17]; however, it remains controversial regarding renal function after RAPN. Moreover, the follow-up period of was short, at approximately 6 months.

In this work, perioperative outcomes and postoperative preservation ratio of the estimated glomerular filtration rate (eGFR) between selective and full clamping groups were compared at 6 and 12 months of follow-up to assess the risks and benefits of selective clamping in RAPN, using propensity-score-matched analysis.

## 2. Materials and Methods

### 2.1. Patient Population

**Figure 1:**
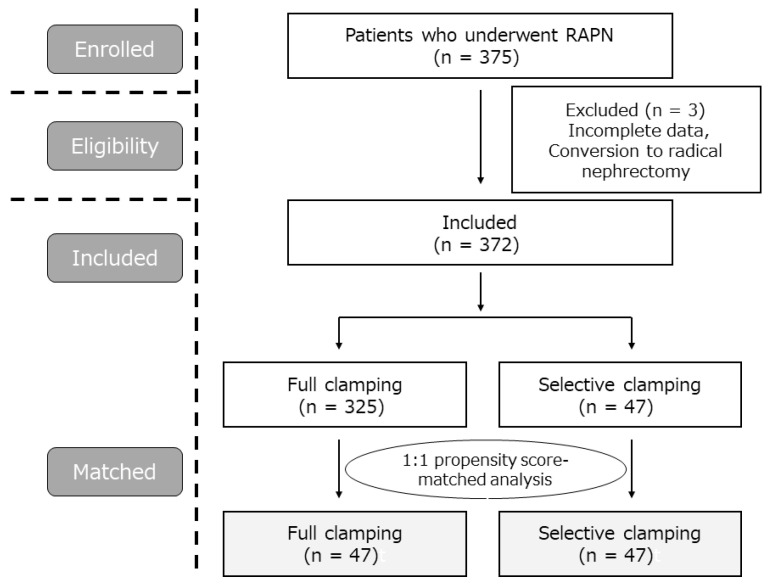
Flowchart of the patients in the study.

In this study, 375 patients who underwent RAPN between July 2010 and March 2021 at our hospital were enrolled. After exclusions due to incomplete data or conversion to radical nephrectomy, 372 patients (full clamping, 325; selective clamping, 47) were included. A 1:1 propensity-score-matched analysis was performed, and 47 patients from each group were evaluated (Figure 1).

### 2.2. Surgery

To construct 3D images for intraoperative navigation, all patients underwent unenhanced abdominal CT as well as four-phase dynamic contrast-enhanced (CE) CT examinations using ultra-high-resolution computed tomography (UHR-CT) or area-detector CT (ADCT). CT images were obtained using a four-phase dynamic CE-CT examination of each CT system. In the present study, ADCT was used for the initial cases (July 2010 to September 2018), and UHR-CT was used for the latter cases (October 2018 to March 2021). All data were obtained via an intraoperative navigation system using TilePro software. 

All RAPN procedures were performed using the da Vinci Xi Surgical System (Intuitive Surgical, Sunnyvale, CA, USA) as previously described [18]. Briefly, the tumor was resected within 2–5 mm of the parenchymal margin. For the inner renorrhaphy layer, the collecting system and large vessels were closed using 3-0 V-Loc sutures. Then, if needed, parenchymal sutures were performed using 2-0 V-Loc, as required. The branch or branches of the renal artery that can supply the renal tumor were identified using UHR-CT or ADCT, and then the selective arterial clamping was performed. When performing the selective clamping in RAPN, the branches of the artery toward the renal tumor were appropriately exposed and clamped with the bulldog. In some cases, intravenous indocyanine green was administered after selective clamping to confirm the region of ischemia using the FIREFLY system (Intuitive Surgical, Sunnyvale, CA, USA). 

All surgeons who completed the Japan-approved da Vinci certification program performed RAPN.

### 2.3. Data Collection

Data were collected preoperatively and at 6 and at 12 months of follow-up. Patient characteristics included age, sex, body mass index (BMI), and American Society of Anesthesiologists (ASA) score. Clinical disease characteristics included tumor side, surgical approach, tumor size, RENAL score [19], presence of a hilar or cystic tumor, and eGFR (mL/min/1.73 m^2^), which was calculated using the Modification of Diet in Renal Disease equation [20]. Surgical parameters included surgical time, console time, WIT, estimated blood loss (EBL), negative surgical margins, pathology, all grades of complications (Clavien–Dindo classification), and the presence of ≥3 complications (Clavien–Dindo classification). Trifecta achievement was defined as WIT ≤ 25 min, no complications, and negative surgical margins [21].

The protocol of this study was approved by the ethics committee of our institution (HM22-176), and the study was performed in accordance with the ethical standards laid down in the most recent version of the Declaration of Helsinki. The need for informed consent from all patients included in this study was waived because of the retrospective design.

### 2.4. Statistical Analyses

Due to inherent differences in baseline patient and disease characteristics between the full and selective clamping groups, we performed 1:1 propensity-score-matched analysis to adjust for imbalances in the confounding factors (age, sex, BMI, ASA score, eGFR, tumor side, approach, tumor size, RENAL score, hilar tumor, and cystic tumor). The propensity scores for each patient were calculated using multivariable logistic regression. Nearest neighbor matching was performed using calipers with a width equal to 0.2 times the standard deviation of the logit of the propensity scores. Intergroup comparisons were performed using a Mann–Whitney U-test, chi-squared test, or Fisher’s exact test. All data were analyzed using IBM SPSS Statistics version 23 (SPSS Japan Inc., Tokyo, Japan), and *p* values < 0.05 were considered statistically significant.

## 3. Results

### 3.1. Clinical Characteristics of the Patients

Patient characteristics, including age, sex, BMI, ASA score, preoperative eGFR, tumor side, surgical approach, tumor size, RENAL score, and the presence of hilar or cystic tumors were compared between the full and selective clamping groups, before and after matching. In the pre-matching cohort, only the tumor side showed a significant difference (*p* = 0.029), whereas no significant differences were observed between the full and selective clamping groups in the post-matching cohort (Table 1). 

### 3.2. Perioperative Outcomes

Following propensity score matching, we compared intergroup perioperative factors. As shown in Table 2, perioperative factors, including surgical time, console time, WIT, EBL, negative surgical margins, pathology, grades of complications, and Clavien–Dindo classification ≥ 3 did not show any differences between the two groups; however, EBL was significantly decreased in the full clamping group than in the selective clamping group (30 vs. 60, *p* = 0.046). Trifecta achievement was observed in 93.6% of patients in the full clamping group and 80.9% in the selective clamping group, with no significant difference (*p* = 0.120).

### 3.3. Renal Functional Outcomes

**Figure 2:**
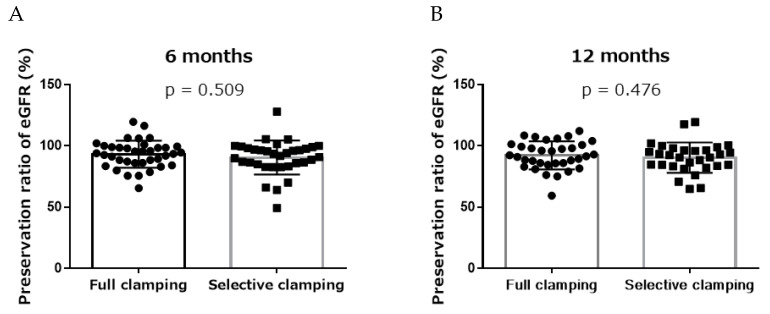
Patients’ functional outcomes. (**A**) 6 months; (**B**) 12 months. Full clamping vs. selective clamping, mean with standard deviation.

There were no significant differences in the preservation ratio of eGFR between the two groups at the 6- or 12-month follow-up (full clamping 94.0% vs. selective clamping 92.7%, *p* = 0.509, and full clamping 92.0% vs. selective clamping 91.6%, *p* = 0.476, respectively) (Figure 2).

**Figure 3:**
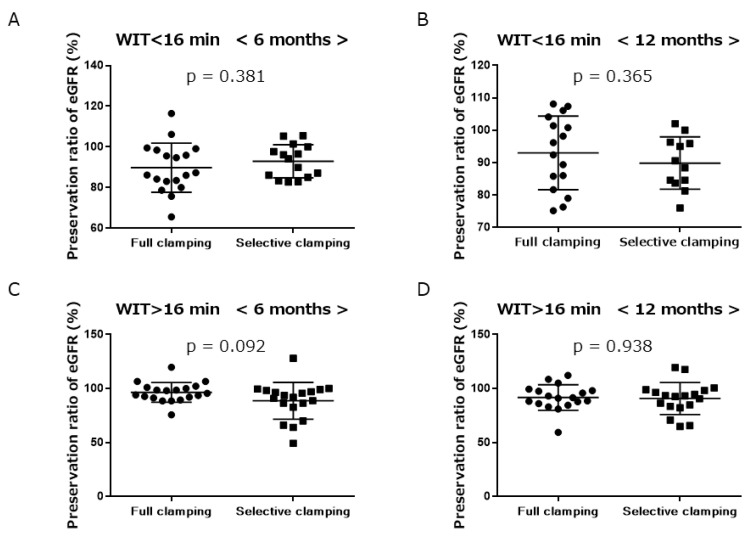
Preservation ratio of eGFR. Category of WIT<16 min ((**A**) 6 months, (**B**) 12 months). Category of WIT>16 min ((**C**) 6 months, (**D**) 12 months). Full clamping vs. selective clamping, mean with standard deviation.

We then assessed the effect of WIT on renal function, regarding clamping. As the median WIT was 16 min in both groups, the preservation ratio of eGFR under or over 16 min of WIT was examined at the 6- or 12-month follow-up. In the category of WIT < 16 min, no significant intergroup differences were observed in the preservation ratio of eGFR at 6 or 12 months (full clamping 86.8% vs. selective clamping 94.1%, *p* = 0.381, and full clamping 94.3% vs. selective clamping 89.5%, *p* = 0.365, respectively) (Figure 3A,B). In the category of WIT > 16 min, no significant intergroup differences were observed in the preservation ratio of eGFR at 6 or 12 months (full clamping 95.6% vs. selective clamping 91.8%, *p* = 0.092, and full clamping 91.3% vs. selective clamping 92.9%, *p* = 0.938, respectively) (Figure 3C,D).

## 4. Discussion

Recently, studies have assessed the effectiveness and utility of selective clamping in RAPN [5,6,7,8,9,10,11,12,13,14,15,16,17]. Regarding renal function after RAPN, while some studies reported a short-term reduction in eGFR in selective clamping [6,7,8,12,13,14], others did not demonstrate improved renal function [9,11]. Moreover, Zhang L. et al. indicated patients undergoing PN with selective clamping had longer surgical time and higher EBL as compared with full clamping [15].

Of the studies, the first randomized controlled trial named EMERALD was reported to assess the impact of super-selective versus global ischemia directly on the operated kidney in RAPN using DMSA scintigraphy [17]. They concluded that super-selective RAPN using near-infrared fluorescence did not provide better renal function preservation than renal artery clamping in non-selected patients at the 6-month follow-up, based on single-surgeon expertise. Additionally, the role of systematic super-selective RAPN was unclear, given the higher vascular injury risks.

Badani et al. evaluated the outcomes of selective arterial clamping in patients with a solitary kidney to remove the influence of a contralateral kidney and compared them to those of full clamping in RAPN [5]. They reported that selective clamping did not appear to provide any functional advantage over full clamping, with similar intra- and postoperative outcomes. 

The results obtained from these recent reports were inadequate for assessing the benefits of renal function in selective clamping in RAPN due to the short follow-up period (approximately 6 months). Moreover, none of the studies focused on WIT when assessing the functional impact of selective clamping in RAPN.

Therefore, we focused on the follow-up period, WIT, and perioperative outcomes and assessed the risks and benefits of selective clamping in RAPN. 

Perioperative outcomes and renal function at 6 and 12 months after RAPN were compared between the selective and full clamping groups after propensity score matching. Our results indicated that among perioperative outcomes, only the EBL was significantly lowered in the full clamping group compared to the selective clamping group. With respect to renal function, no significant differences were observed in the postoperative preservation ratio of eGFR at the 6- or 12-month follow-up between the two groups.

Previous studies have suggested selective clamping might provide some benefits with a longer WIT in RAPN; therefore, we assessed the effect of WIT on renal function in the category of clamping by dividing the two groups with median cut-off values (16 min). In the category of WIT < 16 min, no significant intergroup differences were observed in the preservation ratio of eGFR at 6 or 12 months. Moreover, in the category of WIT > 16 min, no significant intergroup differences were observed in the preservation ratio of eGFR at 6 or 12 months. These results indicated that WIT in selective clamping did not affect renal function after RAPN. 

This study has several limitations. First, this was a retrospective, small, single-institution study that lacked well-designed analyses. In particular, we were unable to perform a technetium-99m diethylene triamine pentaacetic acid (Tc-99m DTPA) diuretic renal scan for precise evaluation of renal function. Second, to adjust for clinical and demographic imbalances, we performed matched-pair analysis, which resulted in a small sample size. Third, although all our surgeons have sufficient experience in performing RAPN, the technical proficiency of the operators may have varied.

## 5. Conclusions

Selective clamping resulted in higher rates of EBL than in full clamping in RAPN. However, selective clamping did not provide any advantage in renal functional over full clamping after RAPN.

## Figures and Tables

**Table 1:** Patients’ clinical characteristics.

	Pre-Matching			Post-Matching		
Median (IQR) or *n* (%)	Full Clamping (*n* = 325)	Selective Clamping (*n* = 47)	*p* Value	Full Clamping (*n* = 47)	Selective Clamping (*n* = 47)	*p* Value
Age	62 (53–70)	60 (51–67)	0.390	58 (46–65)	60 (51–67)	0.222
Sex (%)						
Male	236 (72.6)	33 (70.2)	0.729	33 (70.2)	33 (70.2)	1.000
Female	89 (27.4)	14 (29.8)		14 (29.8)	14 (29.8)	
BMI, kg/m^2^	24 (22–26)	24 (22–26)	0.749	24 (22–27)	24 (22–26)	0.349
ASA score						
1	101 (31.1)	22 (46.8)	0.074	27 (57.4)	22 (46.8)	0.698
2	217 (66.8)	24 (51.1)		19 (40.4)	24 (51.1)	
3	7 (2.2)	1 (2.1)		1 (2.1)	1 (2.1)	
eGFR, mL/min/1.73 m^2^	69.6 (58.9–80.2)	66.2 (51.9–77.1)	0.115	66.5 (56.5–78.2)	66.2 (51.9–77.1)	0.675
Tumor side						
Right	158 (48.6)	31 (66.0)	0.029	30 (63.8)	31 (66.0)	1.000
Left	167 (51.4)	16 (34.0)		17 (36.2)	16 (34.0)	
Approach						
Transperitoneal	156 (48.0)	27 (57.4)	0.275	22 (46.8)	27 (57.4)	0.409
Retroperitoneal	169 (52.0)	20 (42.6)		25 (53.2)	20 (42.6)	
Tumor size, mm	29 (22–37)	30 (22–35)	0.834	25 (20–33)	30 (22–35)	0.193
RENAL score						
4–6	149 (45.8)	15 (31.9)	0.186	21 (44.7)	15 (31.9)	0.136
7–9	155 (47.7)	29 (61.7)		26 (55.3)	29 (61.7)	
10–12	21 (6.5)	3 (6.4)		0 (0)	3 (6.4)	
Hilar tumor	59 (18.2)	10 (21.3)	0.688	8 (17.0)	10 (21.3)	0.794
Cystic tumor	48 (14.8)	8 (17.0)	0.665	5 (10.6)	8 (17.0)	0.552

**Table 2:** Patients’ surgical outcomes.

	Post-Matching		
Median (IQR) or *n* (%)	Full Clamping (*n* = 47)	Selective Clamping (*n* = 47)	*p* Value
Surgical time, min	165 (143–198)	152 (136–180)	0.208
Console time, min	114 (88–132)	102 (90–132)	0.623
WIT, min	16 (12–19)	16 (13–19)	0.738
EBL, mL	30 (15–100)	60 (30–112)	0.046
Negative surgical margins	47 (100)	47 (100)	1.000
Pathology, clear cell carcinoma	13 (27.7)	14 (29.8)	1.000
All grades of complications	2 (4.3)	7 (14.9)	0.158
Clavien–Dindo ≥ grade 3	1 (2.1)	3 (6.4)	0.617
Trifecta achievement	44 (93.6)	38 (80.9)	0.120

## Data Availability

No new data were created or analyzed in this study. Data sharing is not applicable to this article.
